# Positive Association between Endothelium–Platelet Microparticles and Urinary Concentration of Lead and Cadmium in Adolescents and Young Adults

**DOI:** 10.3390/nu13092913

**Published:** 2021-08-24

**Authors:** Chih-Kuo Lee, Charlene Wu, Chien-Yu Lin, Po-Chin Huang, Fung-Chang Sung, Ta-Chen Su

**Affiliations:** 1 Department of Internal Medicine, National Taiwan University Hospital Hsin-Chu Branch, Hsinchu 300, Taiwan; keitheva2009@gmail.com; 2 College of Medicine, National Taiwan University, Taipei 100, Taiwan; 3 Global Health Program, College of Public Health, National Taiwan University, Taipei 100, Taiwan; charlenewu@ntu.edu.tw; 4 Department of Internal Medicine, En Chu Kong Hospital, New Taipei City 237, Taiwan; lin7010@mail2000.com.tw; 5 School of Medicine, Fu Jen Catholic University, New Taipei City 242, Taiwan; 6 Department of Environmental Engineering and Health, Yuanpei University of Medical Technology, Hsinchu 300, Taiwan; 7 National Institute of Environmental Health Sciences, National Health Research Institutes, Miaoli 350, Taiwan; pchuang@nhri.edu.tw; 8 Department of Medical Research, China Medical University Hospital, China Medical University, Taichung 404, Taiwan; 9 Research Center for Environmental Medicine, Kaohsiung Medical University, Kaohsiung 807, Taiwan; 10 Institute of Environmental Health, College of Public Health, China Medical University, Taichung 404, Taiwan; fcsung@mail.cmu.edu.tw; 11 Department of Environmental and Occupational Medicine, National Taiwan University Hospital, Taipei 100, Taiwan; 12 Department of Internal Medicine and Cardiovascular Center, National Taiwan University Hospital, Taipei 100, Taiwan; 13 Institute of Environmental and Occupational Health Sciences, College of Public Health, National Taiwan University, Taipei 100, Taiwan

**Keywords:** lead, cadmium, endothelial microparticles, platelet microparticles

## Abstract

(1) Background: In previous research, higher levels of urine heavy metals, especially lead and cadmium, have been associated with increased cardiovascular risk. However, there is no information linking exposure to heavy metal to endothelial and platelet microparticles (EMPs and PMPs), particularly in the younger population, which are novel biomarkers of endothelial dysfunction. (2) Methods: From a nationwide database, which was incepted in 1992–2000, screening for renal health among Taiwanese school children, a total of 789 subjects were recruited. Cross-sectional analysis was performed to evaluate the association between serum EMPs/PMPs and urine iron, nickel, copper, cadmium, lead, chromium, manganese, and zinc levels in the adolescent and young adult population. (3) Results: After we adjusted the conventional cardiovascular risk factors, CD31+/CD42a− and CD31+/CD42a+ counts, in subjects’ serum, respective markers of EMP and PMP displayed a significant positive dose-response relationship with urinary lead and cadmium levels. Higher quartiles of urine lead and cadmium levels were associated with an increased risk of higher EMPs/PMPs (≥75th percentile) in a multivariate logistic regression model. (4) Conclusion: Higher urinary lead and cadmium concentrations are strongly associated with endothelium–platelet microparticles in this adolescent and young adult population, which could help explain, in part, the mechanism through which heavy metal exposure results in cardiotoxicity.

## 1. Introduction

In recent decades, increased worldwide industrialization, urbanization, and use of fossil fuels have resulted in increasing heavy metal levels in the environment. Human activities such as mining, manufacturing, and fossil fuel burning have directly caused the accumulation of lead in the environment and the human body. Increasingly, adverse health effects attributable to contamination of heavy metals such as copper (Cu), nickel (Ni), lead (Pb), cadmium (Cd), zinc (Zn), manganese (Mn), chromium (Cr), and iron (Fe) are of high concern [1,2]. Moreover, all of them have a high potential to be absorbed, accumulated, and biomagnified in many organs, which in turn can cause various abnormalities and diseases [3].

Lead, as a potential carcinogen, is harmful to the central nervous system, gastrointestinal tract, and kidneys; exposure to Pb may cause birth defects [3,4]. In prior research regarding cardiovascular impact, Pb exposure increased the risk of hypertension; additional research found Pb exposure was associated with a higher incidence of cardiovascular events, including but not limited to coronary artery disease, stroke, and peripheral obstructive arterial disease [5,6]. Furthermore, high blood Pb level was also linked to poor cardiovascular survival outcomes. To identify the underlying mechanisms for the cardiovascular adverse effect of Pb is essential to construct practical preventive strategies [7].

Cadmium is known to accumulate in the proximal tubular cells after entering human bodies and has demonstrated nephrotoxicity and bone homeostasis, which presents as chronic kidney disease and osteoporosis. In addition, Cd can be inhaled through respiratory routes and results in severe lung injuries [8,9,10]. In a previous subclinical study, Cd was found to initiate atherosclerosis and was highly associated with disease progression. Although the exact biochemical mechanism remains unknown, it is evidenced that higher Cd levels accelerate atherosclerotic plaque formation; the plaque accumulation may be attributable to endothelial dysfunction [11].

Recent research reported that urine heavy metal concentrations in Taiwanese population were significantly higher than that detected in other countries; Cd and Pb exposures were the highest in young people [12]. In another study, Cd and Pb exposures in school-aged individuals were associated with statistically significantly higher cardiovascular risks [13]. Thus, evidence of a distinct relation between these metals and CVD risks, especially in young populations, indicates the urgent need for further research. Previously, endothelial microparticles (EMPs) and platelet microparticles (PMPs) were proposed as suitable vascular endothelial dysfunction markers and concentrations of EMPs/PMPS had a strong association with the progression/extent of endothelial injury in cardiovascular disorders [14]. More specifically, previous research stated that human endothelial cells shed CD62E EMP after activation and apoptotic endothelial cells would shed CD31+/CD42a− EMP. Moreover, patients with cardiovascular diseases had higher level CD62P PMP, and CD31+/CD42a+ PMP was shed from apoptotic platelets [15]. Existing studies rarely investigate the interplay among heavy metal toxicity, the cardiovascular system, and subclinical cardiovascular biomarkers; therefore, the objectives of the present research were to evaluate the relation between urinary concentration of heavy metals, with particular focus on Cd and Pb, and biomarkers of cardiovascular microparticles in the Taiwanese adolescent and young adult population.

## 2. Materials and Methods

### 2.1. Study Design, Setting, and Ethics Statement

From 1992 to 2000, a nationwide mass urine screening for renal health was conducted among school children 6 to 18 years of age in Taiwan. The anthropometric status and blood pressure were also measured in all children diagnosed with positive proteinuria or glucosuria twice during the screening examinations (*N* = 103,756). Among these patients, 9227 students were found to have childhood elevated blood pressure (EBP). From 2006 to 2008, we set up a cohort (YOTA, the YOung TAiwanese Cohort Study) according to whether the aforementioned subjects had elevated blood pressure or not in childhood. We sent invitation letters to the parents of eligible students in the Taipei area. After 3–5 days, 12 trained assistants and nurses conducted telephone interviews inviting the subjects with childhood EBP to come to the hospital for a followup health examination. No telephone interview contact was made with normotensive students. Among the 707 subjects with childhood EBP, 303 completed the followup health examinations (Supplementary File S1).

**Figure 1:**
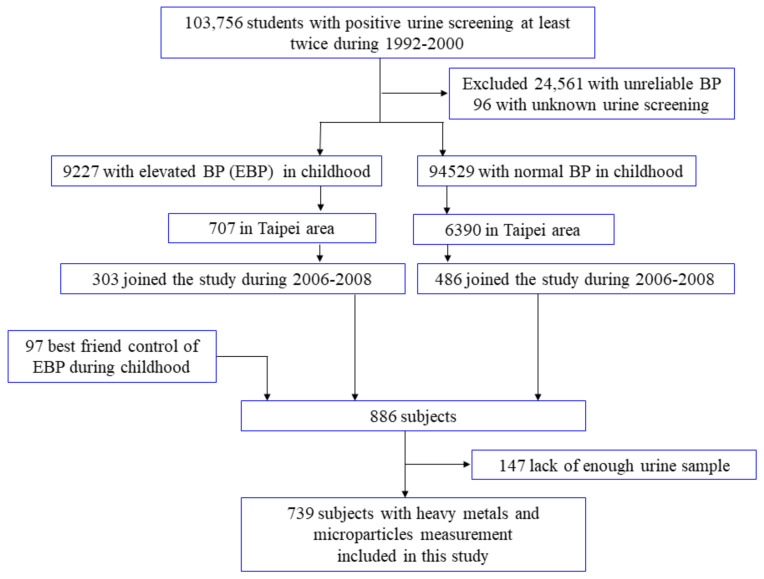
Flow chart of the study population. BP denotes blood pressure and EBP denotes elevated blood pressure.

Among the 94,529 subjects with a normal blood pressure (BP) in childhood with an address, 6390 lived in Taipei. We randomly contacted these sbjects living in Taipei by mail, 5886 of whom did not respond, and 17 refused to participate. Finally, 486 subjects with a normal BP in childhood completed the followup health examinations. With consent, 303 patients with EBP and 486 patients with a normal BP in childhood completed the followup study in the Taipei area. The present research was approved by the Research Ethics Committee of the National Taiwan University Hospital and every participant needed to complete informed consent before enrollment. Among 886 participants, a total of 147 subjects were not available because the urine amount was insufficient to complete all analyses. Finally, 739 subjects were included in this study. The detailed information is also available in previous literature [16,17]. Figure 1 depicted the study flow chart of enrollment, and a total of 739 students were enrolled for the current survey. The definition of school-aged children with positive urine screening results was two sequential positive results for proteinuria, glucosuria, or hematuria within two weeks. Afterward, a third appointment was scheduled, and 96 children were excluded because the abnormality was inconsistent during the third and subsequent followup examinations. The cutoff for positive glycosuria was 1+ or a glucose level of 250 mg/dL (13.9 mmol/L). Albuminuria was defined as a urinary albumin/creatinine ratio ≥30 μg/mg, and hematuria was defined as positive for occult blood using a Hemscomistrix IV urine strip (Ames Division, Miles Lab, Inc., Elkhart, IN, USA) [16,18].

Demographic and social information, such as age, gender, body mass index (BMI), personal habits, and underlying medical history, were recorded and coded in the registry. We used a mercury manometer with adequate cuff sizes to measure blood pressure (both systolic and diastolic blood pressure needed to be recorded), at the same time, heart rate was also checked. We used an autoanalyzer (Technician RA 2000 Autoanalyzer, Bayer Diagnostic, Mishawaka, IN, USA) to measure levels of cholesterol, triglycerides, low-density lipoprotein cholesterol (LDL-C), and glucose in serum. Diabetes mellitus was defined as a fasting serum glucose more than 126 mg/dL, HbA1C > 6.5%, or current use of oral hypoglycemic agents or insulin. Hypertension was defined as current use of antihypertensive drugs or an average blood pressure (BP) greater than 140/90 mmHg for participants ≥18 years, and adolescents younger than 18 having higher blood pressure than ≥95% of peoples’ values in the same population.

### 2.2. Measurement of Urinary Metals and Microparticles

Certain heavy metals in the urine samples were measured as previously described [19,20]. Urinary levels of these eight heavy metals were measured using inductively coupled plasma mass spectrometry (ICP–MS, 7700 series; Agilent Technologies, Inc., Santa Clara, CA, USA). The quality control and method detection limit (MDL) guidelines of this current study follow that of the standard of National Environmental Laboratory Accreditation (NELAC 2003); the protocol was modified from the National Institute of Environmental Analysis (NIEA) PA-103, PA-104, and PA-107, Taiwan EPA (NIEA PA-103, PA-104, PA-107, 2005).

The calibration curve standards ranged from 0.01 ug/L to 50 ug/L for Cr, Mn, Fe, Ni, Cu, Cd, and Pb, 0.05 ug/L to 250 ug/L for Zn (correlation coefficient > 0.995), and one calibration check sample was audited. To perform the quality surveillance for our analyses, one each of blank, spike, duplicate, and quality control samples was performed in every batch of 10 samples. Levels of blank samples of all metals were less than 2 MDL. The spike recovery and quality check samples were within the required ±20%, and duplicate samples were within ±10%. For samples with heavy metal levels below the MDL, a concentration of half the MDL was assigned (Cr: 0.011 μg/L, Mn: 0.017 μg/L, Fe: 0.322 μg/L, Ni: 0.021 μg/L, Cu: 0.276 μg/L, Zn: 0.343 μg/L, Cd: 0.006 μg/L, Pb: 0.007 μg/L). The laboratory was certified by an international laboratory comparison program (G-EQUAS-67) for urinary metals.

EMPs were analyzed using flow cytometers based on a well-established method in the literature [21]. In summary, the microparticles were analyzed in citrated serum by a pair of fluorescent monoclonal antibodies: phycoerythrin-labeled anti CD31 (BD bioscience) and fluorescein isothiocyanate-labeled anti CD42a (BD bioscience). Activation of endothelial cells or platelets were detected using fluorescein isothiocyanate-labeled CD62E or CD62P, respectively. The concentrations of the microparticles were expressed as counts/μL.

### 2.3. Statistical Analysis

The urinary heavy metals levels and serum microparticles were demonstrated as geometric means and interquartile range. We performed the chi-square tests and the Mann–Whitney U test, or the Kruskal–Wallis test to investigate the relationship of the urinary metals concentrations to the categorical variables, because the distribution of both heavy metal and microparticle data were highly skewed.

We took all significant clinical parameters, such as age, gender, and other potential covariates (smoking habit, BMI, SBP and DBP, LDL-C, Log-triglycerides, blood sugar, alcohol consumption, and urine creatinine) into consideration and adjusted for them. Subsequently, we used multiple linear regression to survey any relationship existing between EMPs, PMPs, urinary Pb and Cd, and other heavy metal levels. There was not a clear cutoff value of microparticles to distinguish abnormal from normal population. For clearer demonstration of the relative risk of heavy metal exposure and the risk of higher endothelial–platelets microparticles, we applied the multiple logistic regression method to calculate the odds ratio of higher microparticles (≥75th percentile) for subjects with higher quartiles and per doubling change of urinary concentration of Pb and Cd. We performed all these statistical analyses with SAS software ver. 9.2 (SAS Institute Inc., Cary, NC, USA).

## 3. Results

Table 1 presents the baseline characteristics of the entire study population (294 males and 445 females, N = 739). Compared to their older counterparts, higher urinary levels of Cd, Pb, and Zn were found in adolescents, and subjects with known dyslipidemia had higher urinary levels of Cr and Mn. Males had a higher median concentration of Cd and Mn than females. In addition, concentration of Fe was lower in those with a higher BMI, while Cd concentrations were higher in the cohort with higher BMI. The geometric mean concentrations of the eight heavy metals examined were not different among the other subgroups analyzed.

The geometric mean of urinary concentrations and 95% confidence intervals (CI) of Cd, Pb, Cr, Mn, Fe, Ni, Cu, and Zn were 2.01 ± 2.77 μg/mL, 9.38 ± 17.82 μg/mL, 0.26 ± 0.85 μg/mL, 0.73 ± 1.21 μg/mL, 16.83 ± 41.26 μg/mL, 3.69 ± 14.10 μg/mL, 12.83 ± 23.78 μg/mL and 440.26 ± 316.00 μg/mL, respectively. The mean concentrations and 95% CI of EMPs/PMPs are also listed in Table 2.

We found that the CD31+/CD42a− level increased significantly with levels of Cd, Pb, and Zn, but the Mn level was inversely associated with CD31+/CD42a− counts. There was a positive correlation between CD31+/CD42a+ and Cd and Pb levels (Table 3). Multiple linear regression analysis for every one unit increase in Pb and Cd and the change of microparticles concentration are shown in Table 4. After adjustment for age, gender, and other potential covariates (smoking status, BMI, SBP and DBP, LDL-cholesterol, Log-triglyceride, fasting blood glucose, alcohol consumption, and urinary creatinine), a one unit increase in urinary Cd and Pb levels was positively and significantly associated with changes in microparticles concentration evaluated by CD31+/CD42a−, CD62P, CD31+/CD42a+, and CD14 counts. Table 4 also showed the results if we stratified the patients into active smoker and inactive smoker subgroups. In both subgroups, a one-unit increase in urinary Cd and Pb levels was positively and significantly associated with changes in the concentration of microparticles evaluated based on CD31+/CD42a− and CD31+/CD42a+ counts, which was consistent with the main results. Significant associations between CD62P/CD14 counts and Cd/Pb were only noted in inactive smokers. A similar trend was also found in active smokers, which did not reach statistical significance because of the small number in the active smoker subgroup.

In Table 5, we showed the analytic results of the multivariate logistic regression. Higher quartiles of urine Pb and Cd levels were associated with an increased risk of higher EMPs/PMPs (≥75th percentile). Per doubling change in Pb level, the odd ratios (95% CI) of higher serum EMPs/PMPs levels were 1.01 (1.00–1.02) for CD62E, 1.05 (1.04–1.06) for CD31+/CD42a−, 1.02 (1.01–1.03) for CD62P, 1.01 (1.00–1.02) for CD31+/CD42a+, and 1.02(1.01–1.03) for CD14 counts, respectively. Per doubling change in Cd level, the odd ratios (95% CI) of higher serum EMPs/PMPs levels were 1.37 (1.26–1.49) for CD31+/CD42a−, 1.12 (1.06–1.19) for CD62P, 1.08 (1.02–1.14) for CD31+/CD42a+, and 1.09 (1.03–1.16) for CD14 counts, respectively.

Furthermore, there was a positive trend between CD31+/CD42a−/CD31+/CD42a+ counts and any component of elevated heavy metals. Most importantly, the CD31+/CD42a−/CD31+/CD42a+ counts increased significantly in the Pb > 50th and Cd > 50th subgroups (Table 6).

## 4. Discussion

This cross-sectional study found a strong association between urinary Pb/Cd and cardiovascular microparticles (CD31+/CD42a− and CD31+/CD42a+) in the Taiwanese younger population. We provided clinical evidence linking urinary heavy metals and endothelium–platelet microparticles, which can serve as subclinical markers of endothelial dysfunction. Since the majority of our participants reported that they do not have chronic systemic disease (such as hypertension and diabetes), we were able to observe the association between heavy metals and EMPs/PMPs without interference from cardiovascular diseases (CVD) or risk factors. Our results suggested that exposure to Pb/Cd may activate endothelial and platelet apoptosis, which is positively associated with pathogenesis of atherosclerosis and thus can subsequently increase the risk of cardiovascular morbidities.

Previous research provided evidence that heavy metal exposure is linked to increased risk of CVD and coronary heart disease (CHD). Tellez-Plaza and team conducted a prospective cohort study and provided robust evidence regarding the association between Cd concentrations and cardiovascular diseases. Compared with the 20th percentile of the urine Cd subgroup, the 80th subgroup had hazard ratios (HRs) of 1.43 (95% CI: 1.21–1.70) for cardiovascular mortality and 1.34 (95% CI: 1.10–1.63) for coronary heart disease (CHD) mortality. Additionally, corresponding HRs for incident CVD, CHD, cerebrovascular accident, and heart failure with or without acute decompensation were 1.24 (95% CI: 1.11–1.38), 1.22 (95% CI: 1.08–1.38), 1.75 (95% CI: 1.17–2.59), and 1.39 (95% CI: 1.01–1.94), respectively. Another cross-sectional study conducted in South Korea, also reported a positive association between increasing serum Cd level and higher cardiovascular risk [10]. It is worth noting these findings showed that Cd overexposure is a consistent and strong predictor of the aforementioned circulatory diseases, but the biological mechanism linking heavy metal exposure and adverse health outcomes remains uncertain. Our findings could serve to provide a plausible biological pathway.

In the second National Health and Nutrition Examination Survey (NHANES), Lustberg and Silbergeld (2002) revealed that study participants with blood Pb levels of 20 to 29 μg/dL had a significantly higher relative risk of 1.46 (95% CI: 1.14–1.86) for all-cause death and 1.39 (95% CI: 1.01–1.91) for death from cardiovascular disease compared with subjects whose blood Pb levels were less than 10 μg/dL. The positive association between Pb exposure and increased risk of hypertension is well established; previous research also showed higher Pb concentration was highly associated with hyperlipidemia and atherosclerotic diseases [5,22,23]. Other experimental and human autopsy studies also provided further information that Pb exposure could be linked to atherosclerotic plaque in aorta and had a positive relationship with plaque burden [24,25]. Similarly, a recent study by our group demonstrated that higher residual urinary Pb concentrations was positively associated with increased carotid intima-media thickness (CIMT), as well higher prevalence of metabolic syndrome, especially for adolescents and young adults population in Taiwan [13]. Thus, findings from this current study showing environmental Pb exposure was linked to higher counts of microparticles could serve to bridge the data gap in explaining how Pb subsequently induces increased risk of atherosclerosis and CVDs.

EMPs, a submicron vesicle-like substance, are markers of endothelial dysfunction and released via cell activation and apoptosis [26]. Previous literature proposed many methods to measure these vesicles, since the accumulation could lead to atherosclerotic plaque formation, and found an association with atherosclerosis [27]. Previous studies found significantly higher concentrations of microparticles in high cardiovascular risk populations and in patients with known atherosclerosis and CVD [28,29]. Similarly, plasma endothelial-derived microparticles level were proposed as a balance marker among cell stimulation, proliferation, apoptosis processes, and death [30]. Specifically, platelet derived microparticles stimulated adherence of platelets and endothelial cells to stenotic sites, induced plaque clotting inside lesions, and were strongly linked to inflammatory responses, lipid deposition, and atherosclerosis progression [28]. Significantly higher PMPs were found to be associated with several cardiovascular and cerebrovascular diseases, such as ischemic heart disease, heart failure with and without acute decompensation, hypertension, tachyarrhythmias, thromboembolism events, and subclinical atherosclerosis [31,32]. Therefore, results from our current study may serve to explain, at least partially, that environmental heavy metals such as Pb and Cd have atherogenic potential, as demonstrated by the release and accumulation of microparticles. Furthermore, chronic exposure may result in atherosclerosis and CVD. All of this scientific evidence supports our deduction that the positive association between Pb and increased endothelial and platelet microparticles indicates cardiotoxicity.

Our findings indicated that microparticles from endothelial cells and platelets could provide useful information for screening patients who are at high risk for future cardiovascular events. Detection of the presence and pattern of EMPs/PMPs may facilitate further analysis for the characteristics of endothelial injury [33]. In our present research, a strong association between urinary concentrations of Pb/Cd and CD31+/CD42a−, CD31+/CD42a+ levels was found, which implied that exposure to either heavy metals induce endothelial cell and platelets apoptosis. Further investigations exploring the mechanism through which Pb/Cd influences the aforementioned cell death may be warranted.

We had a few limitations during this study. First, the study population targeted adolescents and the young adult population with an abnormality in urinalysis during school age. Most of these participants lived in the Taipei area, and only less than 1% of the original sample were included in the final analysis; thus, we could not translate a similar pattern to the general population. Although our findings may not be generalized to other populations, we successfully demonstrated that early exposure to heavy metals was positively correlated with EMPs/PMPs counts. Secondly, we did not analyze the interaction between medication use and EMPs/PMPs concentrations, since cardiovascular drugs could potentially confound the association between heavy metal exposures and microparticle formation. However, it is worth noting that such a limitation may not diminish our results, since more than 95% of participants claimed that they do not have any systemic disease (hypertension or diabetes) or medication history. Thirdly, the cross-sectional study was not designed as causality research, therefore, we could not investigate the causality between heavy metals exposure and pre-clinical endothelium–platelet biomarkers. Fourth, the absence of blood lead concentration or other biomarkers (e.g., bone lead concentration) was another limitation. The lead concentrations in plasma and serum are even lower than in urine and Bergdahl, I. A et al. demonstrated that urinary lead concentration was moderately correlated with blood lead concentration [34]. Analyses of urine are also often reliable, but the number of available programs for interlaboratory comparison is smaller [35].

## 5. Conclusions

We reported that higher urinary Pb and Cd concentrations were positively associated with endothelium–platelet microparticles in this adolescent and young adult population. This association remained significant after we adjusted for well-established traditional cardiovascular risk factors. Therefore, Pb/Cd accumulation associated endothelial/platelet activation and apoptosis may cause atherosclerosis pathogenesis with a subsequent increase in risk of cardiovascular morbidities.

## Figures and Tables

**Table 1:** Basic demographics of study participants including geometric means and 95% confidence intervals of creatinine-adjusted urinary heavy metals.

	No.	Cd (μg/L)	Pb (μg/L)	Cr (μg/L)	Mn (μg/L)	Fe (μg/L)	Ni (μg/L)	Cu (μg/L)	Zn (μg/L)
Overall	739	0.22 ± 0.32	1.04 ± 2.07	0.03 ± 0.08	0.08 ± 0.15	1.81 ± 4.2	0.4 ± 1.42	1.36 ± 2.21	47.27 ± 33.63
Age (years)									
12–19	235	0.3 ± 0.38	1.64 ± 2.69	0.03 ± 0.03	0.08 ± 0.09	1.7 ± 2.21	0.43 ± 0.37	1.42 ± 1.26	53.73 ± 36.44
20–30	504	0.18 ± 0.27 ^‡^	0.76 ± 1.64 ^‡^	0.03 ± 0.1	0.08 ± 0.17	1.86 ± 4.86	0.39 ± 1.7	1.33 ± 2.53	44.26 ± 31.83 ^‡^
Gender									
Male	294	0.25 ± 0.33	1.21 ± 2.24	0.03 ± 0.06	0.1 ± 0.19	1.9 ± 3.6	0.42 ± 0.37	1.36 ± 1.18	47.22 ± 33.72
Female	445	0.18 ± 0.29 *	0.78 ± 1.75 ^†^	0.03 ± 0.11	0.06 ± 0.03 ^‡^	1.67 ± 4.98	0.38 ± 2.21	1.36 ± 3.19	47.35 ± 33.55
Smoking habit									
Never	619	0.23 ± 0.32	1.09 ± 2.13	0.03 ± 0.05	0.08 ± 0.09	1.73 ± 3.54	0.43 ± 1.55	1.39 ± 2.36	47.77 ± 33.79
Former	21	0.12 ± 0.15	0.6 ± 1.49	0.1 ± 0.39 ^‡^	0.06 ± 0.03	3.51 ± 10.27	0.27 ± 0.16	1.19 ± 1.08	38.65 ± 20.76
Current	99	0.18 ± 0.29	0.85 ± 1.79	0.02 ± 0.04	0.1 ± 0.34	1.94 ± 5.65	0.26 ± 0.21	1.19 ± 1.12	45.98 ± 34.8
Alcohol Consumption									
Never	656	0.22 ± 0.32	1.03 ± 2.08	0.03 ± 0.05	0.08 ± 0.16	1.79 ± 4.05	0.42 ± 1.5	1.29 ± 1.35	47.42 ± 33.13
Former	13	0.36 ± 0.57	1.4 ± 2.56	0.02 ± 0.02	0.05 ± 0.03	1.4 ± 1.48	0.17 ± 0.1	1.45 ± 1.42	32.75 ± 22.36
Current	69	0.2 ± 0.25	1.05 ± 1.89	0.05 ± 0.22	0.06 ± 0.03	2.07 ± 5.72	0.3 ± 0.24	1.95 ± 5.89	49.05 ± 39.45
Body Mass Index (kg/m^2^)									
<25	122	0.2 ± 0.28	0.91 ± 1.86	0.03 ± 0.09	0.08 ± 0.16	1.88 ± 4.56	0.42 ± 1.55	1.34 ± 2.34	46.62 ± 33.08
≥25	617	0.3 ± 0.45 *	1.7 ± 2.82 ^†^	0.03 ± 0.04	0.08 ± 0.1	1.44 ± 1.29 *	0.33 ± 0.35	1.46 ± 1.31	50.57 ± 36.26
Hypertension									
Yes	231	0.22 ± 0.3	1.02 ± 2	0.03 ± 0.08	0.08 ± 0.15	1.75 ± 3.5	0.41 ± 1.45	1.37 ± 2.25	47.14 ± 33.55
No	508	0.31 ± 0.58	1.54 ± 3.25	0.02 ± 0.03	0.06 ± 0.03 ^†^	3.03 ± 11.49	0.21 ± 0.14 ^†^	1.12 ± 0.86	50.03 ± 35.59
Diabetes Mellitus									
Yes	12	0.22 ± 0.3	1.01 ± 1.99	0.03 ± 0.08	0.08 ± 0.15	1.81 ± 4.23	0.4 ± 1.43	1.35 ± 2.22	46.79 ± 33.16
No	727	0.42 ± 0.75	2.66 ± 4.86	0.02 ± 0.02	0.08 ± 0.03	1.58 ± 1.5	0.36 ± 0.22	1.58 ± 1.48	76.62 ± 48.39
Hyperlipidemia									
Yes	150	0.22 ± 0.31	1.01 ± 2.04	0.03 ± 0.09	0.08 ± 0.17	1.95 ± 4.67	0.43 ± 1.59	1.38 ± 2.4	47.65 ± 33.85
No	589	0.22 ± 0.33	1.15 ± 2.18	0.02 ± 0.03 *	0.07 ± 0.04 *	1.26 ± 1.04 ^†^	0.31 ± 0.23	1.27 ± 1.16	45.78 ± 32.79

Abbreviations: Pb, lead; Cd, cadmium; Zn, zinc; Cu, copper; Ni, nickel; Mn, manganese; Cr, chromium; Fe, iron. * Statistical significance set at *p* < 0.05; † Statistical significance set at *p* < 0.01; ‡ Statistical significance set at *p* < 0.001.

**Table 2:** Geometric mean urinary concentrations and 95% confidence intervals of heavy metals and microparticles.

Metals (µg/L)	Mean ± SD	Max	Min	Median	Q1	Q3	IQR
Cd	2.01 ± 2.77	20.48	0.02	0.71	0.36	3.11	2.75
Pb	9.38 ± 17.82	103.18	0.05	1.35	0.66	5.61	4.95
Cr	0.26 ± 0.85	19.85	0.00	0.13	0.01	0.27	0.25
Mn	0.73 ± 1.21	27.71	0.01	0.58	0.43	0.78	0.35
Fe	16.83 ± 41.26	746.78	1.49	9.58	6.55	14.88	8.33
Ni	3.69 ± 14.10	379.35	0.00	2.48	1.50	3.96	2.46
Cu	12.83 ± 23.78	539.66	1.41	9.27	6.12	13.88	7.76
Zn	440.26 ± 316.00	2470.57	24.62	361.09	211.07	589.96	378.89
**Microparticles**							
CD14	124.64 ± 72.59	1110	0	111.43	80	151.43	71.43
CD62E	263.91 ± 134.99	1391.43	0	235.71	172.86	326.43	153.57
CD62	163.47 ± 113.16	874.29	0	134.29	82.86	211.43	128.57
CD31+/CD42a−	386.42 ± 722.61	11,891.43	0	169.29	61.43	405	343.57
CD31+/CD42a+	11,582.5 ± 18,530.6	129,938.6	0	4180.7	1190.7	12,966.4	11,775.7

Abbreviations: Pb, lead; Cd, cadmium; Zn, zinc; Cu, copper; Ni, nickel; Mn, manganese; Cr, chromium; Fe, iron; SD, standard deviation; IQR, interquartile range.

**Table 3:** Mean and standard error of urinary heavy metals’ concentrations across categories of endothelial and platelet microparticles in linear regression models (*N* = 739).

	CD31+/CD42a− Counts, µL				
	Quartile 1	Quartile 2	Quartile 3	Quartile 4	
	≤61.43	61.43–169.29	169.29–405	>405	***p*-Trend**
**Metals (µg/L)**	177	191	187	184	
Cd	0.86 ± 1.09	1.32 ± 2.09	2.07 ± 2.73	3.78 ± 3.56	<0.0001
Pb	1.56 ± 2.24	3.86 ± 10.37	9.82 ± 17.57	22.20 ± 24.36	<0.0001
Cr	0.23 ± 0.48	0.28 ± 1.46	0.25 ± 0.49	0.30 ± 0.45	0.5932
Mn	0.96 ± 2.21	0.65 ± 0.68	0.66 ± 0.72	0.65 ± 0.43	0.044
Fe	19.04 ± 40.23	15.43 ± 39.57	18.84 ± 58.70	14.11 ± 12.71	0.4811
Ni	4.99 ± 28.39	3.02 ± 2.78	3.28 ± 2.92	3.56 ± 2.80	0.3452
Cu	10.52 ± 8.77	11.26 ± 19.59	14.62 ± 39.99	14.86 ± 12.95	0.1498
Zn	392.39 ± 270.32	413.60 ± 299.47	447.54 ± 303.53	506.61 ± 371.79	0.0003
	**CD31+/CD42a+ Counts, µL**				
	**Quartile 1**	**Quartile 2**	**Quartile 3**	**Quartile 4**	***p*** **-Trend**
	≤1190.71	1190.07–4180.71	4180.7–12,966.43	>12,966.43	
**Metals (µg/L)**	182	185	191	181	
Cd	1.07 ± 2.06	2.01 ± 2.74	2.41 ± 2.95	2.56 ± 2.98	<0.0001
Pb	3.79 ± 12.38	9.12 ± 17.67	11.23 ± 19.71	13.32 ± 19.16	<0.0001
Cr	0.23 ± 0.49	0.25 ± 0.44	0.23 ± 0.41	0.35 ± 1.52	0.2016
Mn	0.82 ± 1.02	0.82 ± 2.05	0.63 ± 0.35	0.63 ± 0.75	0.1546
Fe	16.91 ± 24.55	17.10 ± 37.67	18.16 ± 55.88	15.05 ± 40.26	0.7724
Ni	5.14 ± 28.02	3.07 ± 2.37	3.36 ± 3.02	3.22 ± 2.75	0.2841
Cu	15.32 ± 43.89	11.32 ± 11.45	12.86 ± 11.95	11.84 ± 9.34	0.2222
Zn	429.46 ± 284.31	429.62 ± 316.57	464.24 ± 331.44	436.70 ± 329.94	0.5894

Abbreviations: Pb, lead; Cd, cadmium; Zn, zinc; Cu, copper; Ni, nickel; Mn, manganese; Cr, chromium; Fe, iron.

**Table 4:** Multiple linear regression analysis for every one unit increase in Pb and Cd and the change in microparticle concentration.

	EMPs		PMPs		
	CD62Ecounts	CD31+/CD42a−_countsUL	CD62Pcounts	CD31+/CD42a+_countsUR	CD14counts
Cd, μg/L	3.03 ± 1.91	59.67 ± 7.03	6.77 ± 1.62	1007.06 ± 259.22	3.24 ± 1.05
*p*	0.1133	<0.0001	<0.0001	<0.0001	0.0022
Pb, μg/L	0.56 ± 0.30	10.04 ± 1.08	1.15 ± 0.25	176.17 ± 40.13	0.71 ± 0.16
*p*	0.0618	<0.0001	<0.0001	<0.0001	<0.0001
**Active smokers (N = 99)**					
Cd, μg/L	0.29 ± 4.51	108.22 ± 22.22	5.79 ± 4.24	1732.68 ± 676.46	3.34 ± 2.50
*p* value	0.949	<0.001	0.175	0.012	0.185
Pb μg/L	−0.12 ± 0.73	15.32 ± 3.70	0.32 ± 0.69	454.89 ± 102.18	0.97 ± 0.39
*p* value	0.865	<0.001	0.645	<0.001	0.016
**Inactive smokers (N = 638)**					
Cd, μg/L	3.30 ± 2.12	54.50 ± 7.36	6.91 ± 1.72	1012.36 ± 276.46	3.51 ± 1.13
*p* value	0.120	<0.001	<0.001	<0.001	0.002
Pb, μg/L	0.67 ± 0.33	9.95 ± 1.12	1.22 ± 0.27	153.08 ± 42.91	0.70 ± 0.17
*p* value	0.043	<0.001	<0.001	<0.001	<0.001

Adjusted for age, sex, BMI, systolic and diastolic BP, LDL-cholesterol, Log triglycerides, fasting glucose, and alcohol habit, and urine creatinine.

**Table 5:** Odds ratio and 95% confidence intervals of adjusted markers of microparticles across categories of Pb and Cd in multiple logistic regression models (N = 739).

	Range (ug/L)	N	≥75th Percentile EMPs or PMPs				
			EMPs		PMPs		
			CD62E Counts	CD31+/CD42a−_Counts UL	CD62P Counts	CD31+/CD42a+_Counts UR	CD14 Counts
**Pb**							
Quartile 1	≤0.657	184	1 (Ref)	1 (Ref)	1 (Ref)	1 (Ref)	1 (Ref)
Quartile 2	0.657–1.345	186	0.85 (0.50, 1.45)	1.36 (0.69, 2.69)	1.09(0.66, 1.80)	0.52 (0.29, 0.95)	1.05 (0.59, 1.87)
Quartile 3	1.345–5.610	185	1.05 (0.63, 1.76)	2.72 (1.45, 5.09)	0.75 (0.44, 1.27)	1.56 (0.95, 2.57)	1.40 (0.80, 2.44)
Quartile 4	>5.610	184	1.61 (0.97, 2.65)	13.22 (7.12, 24.55)	1.68 (1.02, 2.78)	2.10 (1.28, 3.46)	3.49 (2.06, 5.91)
*p*-trend			0.0047	<0.0001	0.0140	<0.0001	<0.0001
Per doubling change			1.01 (1.00, 1.02)	1.05 (1.04, 1.06)	1.02 (1.01, 1.03)	1.01(1.00,1.02)	1.02 (1.01, 1.03)
**Cd**							
Quartile 1	≤0.360	184	1 (Ref)	1 (Ref)	1 (Ref)	1 (Ref)	1 (Ref)
Quartile 2	0.360–0.710	186	0.75 (0.44, 1.30)	1.74 (0.85, 3.54)	1.35 (0.80, 2.29)	0.53 (0.30, 0.95)	0.85 (0.47, 1.51)
Quartile 3	0.710–3.111	185	1.29 (0.78, 2.13)	4.76 (2.49, 9.09)	1.05 (0.61, 1.81)	1.20 (0.73, 1.99)	1.76 (1.04, 2.99)
Quartile 4	>3.111	184	1.14 (0.68, 1.92)	15.72 (8.18, 30.20)	2.00 (1.19, 3.38)	1.72 (1.04, 2.85)	1.99 (1.17, 3.38)
*p*-trend			0.0573	<0.0001	0.0013	0.0004	<0.0001
Per doubling change			1.05 (0.99, 1.11)	1.37 (1.26, 1.49)	1.12 (1.06, 1.19)	1.08 (1.02, 1.14)	1.09 (1.03, 1.16)

Adjusted for age, sex, BMI, systolic and diastolic BP, LDL, Log triglycerides, fasting glucose, smoking and alcohol habit, and urine creatinine.

**Table 6:** Mean (S.E.) of microparticles in different Pb and Cd subgroups in the multiple linear regression model.

	CD31+/CD42a−				CD31+/CD42a+			
	Mean	SE	*p* Value	*p* for Trend	Mean	SE	*p* Value	*p* for Trend
Pb ≤ 50th and Cd ≤ 50th	218.41	40.56	Reference	<0.001	8647.03	1448.90	Reference	<0.001
Pb > 50th and Cd ≤ 50th	270.09	73.79	0.697		7541.92	2605.26	1.000	
Pb ≤ 50th and Cd > 50th	334.17	72.93	1.000		13,375.92	2635.98	0.406	
Pb > 50th and Cd > 50th	579.29	40.79	<0.001		17,363.41	1457.19	<0.001	

Adjusted for age, sex, BMI, systolic and diastolic BP, LDL-C, Log triglycerides, fasting glucose, smoking and alcohol habit, and urine creatinine.

## Data Availability

All relevant data are within the manuscript.

## Supplementary Materials

The following are available online at https://www.mdpi.com/article/10.3390/nu13092913/s1, File S1: The details of the study population and data collection are described in the supplementary material file.
