# Supplementary material for: Positive Association between Endothelium–Platelet Microparticles and Urinary Concentration of Lead and Cadmium in Adolescents and Young Adults
Source: Nutrients. 2021 Aug 24;13(9):2913. doi: 10.3390/nu13092913 (PMC8468016; doi:10.3390/nu13092913)
Supplement: Supplementary file 1 [file nutrients-13-02913-s001.zip › nutrients-1275081-supplementary.pdf]

Supplementary material

Study population and data collection

From 1992 to 2000, approximately 2,615,000 to 2,932,000 school-age children in grades 1 to 12 received an annual urine screening of urine strip by the Chinese Foundation of Health in Taipei, Taiwan. Subjects with abnormal results from two tests for proteinuria, glycosuria, or hematuria underwent a third urine screening test and a general health check-up. A total of 103,756 school children received the health check-ups and the third urine screen. Among these children, 9,227 had elevated blood pressure and 94,529 had normal blood pressure.

From 2006 to 2008 we established a cohort, the YOung TAiwanese Cohort (YOTA) study, based on students with and without childhood EBP, selected from the 1992–2000 urine mass screening population. In the follow-up, we mailed invitation letters to eligible students in the Taipei area. After 3–5 days, 12 trained assistants and nurses conducted telephone interviews inviting those subjects with childhood EBP to come in for a follow-up health examination. No telephone interview contact was made with normotensive students. Among the 707 subjects with EBP in childhood, 303 completed the follow-up health examinations, giving a response rate of 42.9%. Among the

6,390 subjects with normal BP in childhood, 486 completed the follow-up health examinations, giving a response rate of 7.6%. In order to differentiate the effects of environment on age of exposure, we recruited 97 subjects as “best friend controls” in the cohort follow-up period. A total of 886 subjects were included in this study. Physical check-ups were given after written informed consent. All methods in this study were performed in accordance with the relevant guidelines and approved by the Research Ethics Committee of at the National Taiwan University Hospital. A flow chart of this study is shown in Figure 1. Among 886 participants, urine samples for testing heavy metals were unavailable in 147 subjects. Finally, 739 subjects were included in this study. The Fig. 1 depicted the study flow chart of enrollment and a total of 739 students were enrolled for current survey.
